# Clinical sub-phenotypes of co-occurring metabolic syndrome and pre-frailty and their associated factors in older adults: findings from Whitehall II study

**DOI:** 10.3389/fmed.2026.1909752

**Published:** 2026-09-08

**Authors:** Steve Kevin Njouonkep Sime, Mélanie Pétéra, Léopold K. Fezeu, Blandine Comte, Estelle Pujos-Guillot

**Affiliations:** 1 Université Clermont Auvergne, INRAE, UNH, Plateforme d'Exploration du Métabolisme, MetaboHUB Clermont, Clermont-Ferrand, France; 2 Sorbonne Paris Cité Epidemiology and Statistics Research Center (CRESS), Nutritional Epidemiology Research Team (EREN), INSERM U1153, INRAE U1125, Cnam, University of Paris 13, Bobigny, France

**Keywords:** co-occurring conditions, frailty, healthy ageing, metabolic sybdrome, multimorbidity (co-morbidity), personnalised medicine, sub-phenotypes

## Abstract

**Background:**

There is emerging evidence that frailty, and its early stage, pre-frailty, are closely linked to metabolic changes, particularly in response to environmental factors such as lifestyle and diet. There is therefore a major interest in exploring these links, particularly with regard to the highly prevalent metabolic syndrome (MetS) and heterogeneity in phenotypes due to the multicriteria nature of both syndromes. In order to structure this heterogeneity, this study aimed to identify and characterize novel sub-phenotypes of MetS and (pre-)frailty co-occurrence in older adults, as well as to examine the associated clinical, psychosocial, and behavioral factors.

**Methods:**

Data from the Whitehall II cohort (2015–2016 wave), including 3,398 participants aged ≥60 years, were used. A data-driven approach was applied using agglomerative hierarchical clustering based on selected clinical parameters defining MetS (harmonized consensus definition) and pre-frailty (Fried phenotype). After deriving and describing the resulting sub-phenotypes (SPs)**,** their associated factors were examined using a multinomial logistic regression model that simultaneously included a broad set of biological, sociodemographic, lifestyle, and health variables.

**Results:**

Four clinical SPs (intra-sex concordance >87%) emerged from this reclassification, each characterized by distinct patterns of cardiometabolic and functional parameters. The first profile (SP1 31.7%) showed the most favorable cardiometabolic parameters and superior functional performance, although handgrip strength and physical activity levels were slightly lower; it was associated with more favorable socio-demographic, lifestyle, and psychosocial characteristics. A second profile (SP2, 28.7%) displayed cardiometabolic burden, particularly hypertension, with still preserved physical functional characteristics. The two last profiles, SP3 (6.4%) characterized by slow gait, lower physical activity, and exhaustion despite largely preserved metabolic parameters, whereas SP4 (33.2%) showing abdominal obesity, hypertriglyceridemia, hyperglycemia, low HDL-cholesterol, and functional impairments, were associated with older age and male sex. Lower fruit and vegetable intake and poorer physical quality-of-life were associated to SP2 and SP4. Depression and poor mental health markedly increased the likelihood of belonging to SP3.

**Conclusions:**

By structuring heterogeneity into clinically interpretable sub-phenotypes, these findings provide a framework for multidimensional assessment of complex age-related conditions and generate hypotheses regarding potential combinations of underlying metabolic, functional, and psychosocial mechanisms.

## 1 Introduction

Global aging is reshaping health-care priorities as older adults increasingly accumulate multiple chronic conditions (1). Among the processes underlying this burden, metabolic abnormalities and functional decline stand out as major drivers of morbidity and mortality in later life (2–4). In particular, frailty is one of the most critical age-related vulnerability syndromes that reflects diminished physiological reserve and heightened vulnerability to stressors (5). Although no single definition exists, it is increasingly recognised as a multidimensional construct encompassing physical, psychological and social domains. Fried's phenotype defines frailty as the presence of at least three out of five criteria (unintentional weight loss, low grip strength, exhaustion, slow gait speed and low physical activity), while one or two criteria indicate a pre-frail state that represents an intermediate and often reversible stage on frailty continuum (5). Frailty is a well-established predictor of adverse outcomes such as falls, hospitalisation, disability and mortality (6). Pre-frailty, in contrast, is far more common and represents an earlier and more reversible stage, making it a critical target for prevention (7, 8).

There is now strong evidence that metabolic changes play a key role in the aging process, particularly in response to environmental factors such as lifestyle and diet (9). Given these close connections, interest in exploring the relationship between metabolic disorders and frailty in aging has been steadily increasing. In particular, focus has been given to the metabolic syndrome (MetS) and frailty relationship that poses major threats to healthy aging and independence (10, 11).

MetS, defined by the presence of at least three out of five metabolic disorders (abdominal obesity, hypertension, hyperglycemia, blood low HDL-cholesterol and elevated triglycerides), becomes more prevalent with age and substantially increases the risk of cardiovascular disease, type 2 diabetes and related mortality (12).

MetS and (pre-)frailty frequently coexist, with a recent meta-analysis reporting a prevalence of 20% for frailty and 45% for pre-frailty, among older adults with MetS (13). They share common underlying pathophysiological mechanisms such as systemic inflammation, insulin resistance and oxidative stress, which can accumulate and potentiate each other, thereby exacerbating adverse outcomes (11, 14). Although evidence suggests a bidirectional relationship between the two syndromes, most studies support a predominant pathway whereby MetS and its components increase the risk of pre-frailty and frailty, with frailty itself emerging as the strongest predictor of negative health outcomes (11, 15–19). Despite this close interconnection and their multicriteria nature that identifies individuals at particularly high risk, most studies examined MetS and (pre-)frailty in isolation and relied on dichotomous definitions (presence vs. absence). Such a binary approach fails to capture the heterogeneity of these syndromes and may obscure intermediate or mixed phenotypes. Furthermore, it implicitly assumes that all diagnostic criteria contribute equally to risk, overlooking the possibility that specific combinations of abnormalities may drive distinct trajectories. An integrated, data-driven approach is therefore needed to provide a more comprehensive understanding.

Unsupervised clustering techniques have recently been employed to delineate clinically meaningful subgroups within heterogeneous diseases. In the study of MetS, unsupervised clustering approaches have identified distinct MetS endotypes characterized by different cardiometabolic and clinical profiles, highlighting the heterogeneity underlying MetS beyond conventional diagnostic definitions (20). Similarly, clustering and latent class approaches applied to frailty revealed distinct subtypes with different functional, physical, and multidimensional characteristics, supporting the existence of heterogeneous frailty phenotypes among older adults (21, 22). More recently, Yang et al. (2025) applied K-means clustering in hospitalized frail older adults and identified clinically distinct frailty subgroups associated with different survival outcomes, further supporting the relevance of unsupervised approaches in characterizing frailty heterogeneity (23). However, while unsupervised approaches have been separately applied to MetS and frailty, their joint application to the defining criteria of both conditions remains largely unexplored. It may be possible to delineate clinically meaningful high-risk subgroups that reflect different combinations of metabolic and functional impairments. Identifying such sub-phenotypes could help clinicians stratify risks more accurately and tailor preventive interventions. This study aimed to apply an unsupervised approach jointly combining the defining criteria of MetS and (pre-)frailty in older adults (with a specific focus on pre-frailty as an earlier and more actionable stage of the frailty continuum), in order to identify novel clinical sub-phenotypes of their co-occurrence beyond traditional binary classifications. We further sought to characterize these sub-phenotypes in terms of their clinical, sociodemographic, lifestyle, and psychosocial profiles.

## 2 Materials and methods

### 2.1 Study population

This cross-sectional study was conducted on the 2015–2016 wave of the Whitehall II study, a prospective cohort initiated in 1985.

The original cohort included 10,308 civil servants (men and women), aged 35 to 55 years, with the primary objective of investigating the social determinants' inequalities and their impact on health (24). Data collection alternated between questionnaire-only phases and those including clinical examinations. Participant consent and ethical approval were renewed at each follow-up to ensure compliance with ethical standards.

The 2015–2016 wave was selected as the last with complete questionnaire and clinical data available at the time of analysis, and because it included participants aged ≥60, making it suitable for examining age-related conditions. This wave included both self-reported and clinical data.

### 2.2 Outcome

In this study, which focused on the co-occurrence of MetS and frailty syndromes, we restricted our analysis of frailty to pre-frailty as the outcome of interest, since it represents an earlier and more clinically and metabolically reversible state than frailty. Pre-frailty thus constitutes an intermediate risk profile that is both highly prevalent and more amenable to preventive interventions (7, 8).

The MetS-pre-frailty co-occurrence was defined as the simultaneous presence of MetS and pre-frailty, defined following the consensus threshold definition for MetS (12), and Fried's phenotype criteria for pre-frailty (5).

The MetS was evaluated on the basis of the following components: (i) Abdominal obesity, defined by waist circumference measurements; (ii) Low HDL-cholesterol (HDL-chol), determined by blood HDL- cholesterol levels and/or the use of lipid-lowering medications; (iii) Hypertriglyceridemia, defined by blood triglyceride (TG) levels and/or the use of lipid-modifying medications; (iv) Dysglycemia, assessed through the measurement of fasting blood glucose levels and/or the use of glucose-lowering medications; (v) Hypertension, based on elevated systolic and/or diastolic blood pressure levels and/or the use of antihypertensive medications.

The assessment of pre-frailty was conducted using the five following criteria: (i) Exhaustion, determined based on responses to the Centre for Epidemiologic Studies-Depression (CES-D) scale (25); (ii) Low physical activity, defined as energy expenditure in kcal/week, as previously described in the cohort (26); (iii) Slow walking speed, assessed by the time required to walk 2.4 m; (iv) Low grip strength, measured using the Smedley hand grip dynamometer; (v) Weight loss (shrinking), originally defined in the context of frailty as an unintentional weight loss of at least 5% over the previous year, was adapted as did in the Women's Health Aging Study I (27). Detailed descriptions of the assessment processes (including the thresholds) can be found in Supplementary 1.

In total, fifteen clinical variables were used to define the two syndromes: nine defining MetS (waist circumference, fasting blood glucose levels, use of hypoglycaemic medication, blood HDL-chol levels, blood TG levels, use of lipid-lowering medication, systolic blood pressure (SBP), diastolic blood pressure (DBP), and use of antihypertensive medication), and six defining pre-frailty (two CES-D items, caloric expenditure, walking time, percentage of weight variation, and grip strength).

### 2.3 Subject selection

Prior to the 2015–2016 wave, 1,799 individuals died, and 2,877 did not respond during this period. In addition, further exclusions were applied in order to ensure the validity of the analysis. Participants with incomplete data on the criteria used to define the two syndromes (*n* = 1,805) were removed. Additionally, to minimize the confounding effects of multimorbidity and account for the potential impact of severe medical conditions on clinical and metabolic phenotypes, 313 individuals diagnosed with dementia, coronary heart disease, depression, Parkinson's disease, end-stage renal failure, chronic obstructive pulmonary disease, or stroke were excluded. Finally, given the specific focus of the study on pre-frailty, 116 participants who were classified as frail were also excluded. This process resulted in a final analytical sample of 3,398 participants (see flowchart in Supplementary 2).

### 2.4 Statistical analysis

Within this final analytical sample (*n* = 3,398), complete data were available for all outcome variables, as participants with missing values on these variables had been excluded during the sampling process. For the other variables missing data were below 5%. When describing the study sample (Tables 1, 2) categorical variables are presented as absolute frequencies and percentages, including a separate category for missing values. Continuous variables are reported as means ± standard deviation (SD) or as medians with interquartile ranges (IQR, 25th-75th percentiles), depending on the normality of their distribution assessed by the Shapiro–Wilk test.

**Table 1:** Test-values of variables used for HCA, for each sub-phenotype.

Characteristics	Sub-phenotype 1		Sub-phenotype 2		Sub-phenotype 3		Sub-phenotype 4	
	Test-value	*p*-value	Test-value	*p*-value	Test-value	*p*-value	Test-value	*p*-value
MetS variables								
Waist circumference	−14.6	<0.001	0.71	0.4	0.9	0.4	12.7	<0.001
Blood triglycerides levels	−7.4	<0.001	−3.2	0.001	−0.01	0.9	10.1	<0.001
Blood HDL-cholesterol levels	−12.3	<0.001	−29.3	<0.001	1.4	0.1	39.1	<0.001
Blood glycemia	−9.06	<0.001	−5.3	<0.001	−0.45	0.6	13.9	<0.001
High Blood pressure	−49.9	<0.001	19.8	<0.001	1.11	0.3	27.8	<0.001
Pre-frailty variables								
Walking speed	−6.22	<0.001	−7.5	<0.001	3.27	0.005	11.4	<0.001
Handgrip strength	−3.002	0.003	5.6	<0.001	−1.02	0.3	−1.99	0.04
Weight variation	−1.65	0.1	1.64	0.1	−0.09	0.9	0.03	0.9
Physical activity	−6.15	<0.001	15.7	<0.001	−2.76	0.006	−7.88	<0.001
Exhaustion	−9.21	<0.001	−9.47	<0.001	54.8	<0.001	−10.38	<0.001

**Table 2:** Clinical and physical characteristics of participants in the Whitehall II cohort study (*n* = 3,398) according to the sub-phenotypes' classification.

Characteristics	MetS-pre-frailty co-occurrence				
	Sub-phenotype 1	Sub-phenotype 2	Sub-phenotype 3	Sub-phenotype 4	*p*-value^a^
Number of subjects (%)	974 (28.7)	1,078 (31.7)	219 (6.4)	1,127 (33.2)	
Weight (kg)^b^	72.8 (12.4)	78.1 (14.9)	77.6 (14.2)	81.4 (14.5)	**<0**.**0001**
BMI (kg/m^2^)^c^	24.7 (3.4)	26.9 (4.3)	27.3 (4.7)	27.9 (4.3)	**<0**.**0001**
Waist circumference (cm)^b^	91.6 (10.5)	97.1 (12.2)	97.5 (12.5)	101 (11.4)	**<0**.**0001**
Fasting blood glucose (mM)^c^	5.0 (4.70–5.30)	5.1 (4.80–5.50)	5.1 (4.70–5.60)	5.3 (4.90–5.80)	**<0**.**0001**
Systolic blood pressure (SBP, mmHg)^b^	116.3 (9.1)	134 (15.7)	125.5 (15.6)	128.3 (16.2)	**<0**.**0001**
Diastolic Blood pressure (DBP, mmHg)^b^	67.7 (7.3)	75.9 (10.4)	70.8 (9.9)	71.7 (10.3)	**<0**.**0001**
Triglycerides (mM)^c^	0.90 (0.70–1.20)	1.0 (0.80–1.30)	1.0 (0.80–1.40)	1.1 (0.80–1.60)	**<0**.**0001**
HDL- cholesterol (mM)^c^	1.70 (1.40–2.00)	1.60 (1.40–2.00)	1.5 (1.30–1.90)	1.4 (1.20–1.80)	**<0**.**0001**
Gait speed (seconds)^c^	2.10 (1.84–2.39)	2.22 (1.94–2.54)	2.32 (2.05–2.63)	2.25 (1.97–2.6)	**<0**.**0001**
Handgrip test measure (kg)^c^	34 (26–41)	33 (25–41)	33 (25–40)	34 (27–40)	<0.2
Energy expense of physical activity (Kcal)^c^	942 (368–1,688)	998 (341–1,979)	740 (236–1,360)	751 (227–1,517)	**<0**.**0001**
Weight variation (%)^c^	−2.77 (0.00 - + 1.87)	−0.15 (−2.87 - + 2.31)	−2.69 (−0.36 - + 2.28)	+0.22 (−2.48 − +2.62)	0.06

a ANOVA (or non-parametric equivalent Kruskal–Wallis). Significant *p*-values (≤0.05).

b Mean (SD).

c Median (25−75^Pct^).

#### 2.4.1 Existing clinical phenotypes

The clinical status of participants based on the presence or absence of MetS and pre-frailty leads to a large number of theoretically possible clinical phenotypes, due to the combination of the different clinical criteria defining the syndromes. Specifically, the five binary components of MetS and pre-frailty generate 16 and 15 distinct combinations, respectively, yielding a total of 240 theoretical combinations when considering their co-occurrence. We refer to these as *clinical phenotypes*—i.e., all potential configurations defined by established clinical thresholds.

This high dimensionality poses several challenges. Firstly, many of these theoretical clinical phenotypes may not be observed in the actual sample and thus offer limited value for interpretation. Secondly, even when observed, some combinations may represent very small groups, limiting statistical power and clinical applicability. In addition, such rule-based classifications may overlook meaningful patterns present in the data that do not align perfectly with predefined thresholds.

To overcome these limitations and to uncover clinically meaningful patterns, we employed an unsupervised data-driven approach. Specifically, a hierarchical agglomerative clustering (HAC) was applied to the full analytical sample, using variables defining both syndromes' clinical criteria. This approach allows the identification of *clinical sub-phenotypes*, defined here as empirically derived clusters of individuals with similar metabolic and frailty profiles.

The goal of this method was twofold: (i) to explore the phenotypic structure of this elderly population by moving beyond clinical thresholds of MetS and pre-frailty, (ii) to focus on the most prevalent or internally coherent phenotypes in order to describe their clinical characteristics and main associated factors. This strategy aims to support future research in personalized medicine by providing a more nuanced, data-driven classification of metabolic–pre-frailty profiles.

##### 2.4.1.1 Hierarchical agglomerative clustering (HAC)

A HAC analysis, an unsupervised data analysis method that enables to group individuals and/or variables into clusters based on their similarities, was conducted. This approach facilitates the identification of unsupervised emerging sub-phenotypes according to their shared characteristics and the discovery of intrinsic structures within the data without the necessity of prior knowledge of the grouping patterns. The HAC analysis was conducted using FactoMineR (28), subsequent to data standardization, i.e., mean-centering and scaling. HAC was then applied using the Euclidean distance as a measure of proximity between individuals and the Ward's method as the clustering linkage criterion, a standard approach that minimizes within-cluster variance in Euclidean space for standardized numerical data (29). Following the implementation of HAC, a dendrogram was generated and the optimal number of clusters was evaluated by combining visual inspection of the dendrogram tree structure with quantitative internal validation metrics across candidate partitions ranging from k = 2 to 10. Partition quality was assessed using three complementary criteria: the Calinski-Harabasz index, the total within-cluster sum of squares (Elbow method), and the average silhouette width. Internal validation and cluster stability were evaluated using bootstrap resampling (B = 1,000 iterations) via the fpc R package. Stability was quantified using the mean Jaccard similarity index for each derived sub-phenotype, with indices >0.75 defining highly stable clusters and values >0.60 representing acceptable stability (30, 31).

##### 2.4.1.2 Variable selection for the hierarchical agglomerative clustering

HAC has been shown to be sensitive to correlations, particularly in cases where the number of variables is limited, as in the present study, where the dataset included 15 variables for both syndromes. Previous understanding of the pathophysiology of MetS and pre-frailty allowed us to hypothesize specific strong correlations between variables, which might have influenced the clustering process, potentially resulting in an over-representation of certain criteria. For instance, dyslipidemia-related components, such as hypertriglyceridemia or low HDL-chol and lipid lowering medication (which is identical for both), are linked, potentially resulting in an artificial overweight of these characteristics in HAC. Given the sensitivity of this method to excessive correlations, we systematically examined these interdependencies to determine whether reducing the number of variables was necessary. The objective was to ensure that the clustering process remained clinically relevant while avoiding distortions caused by redundant information.

A further significant challenge pertained to the unequal distribution of variables across the two syndromes (nine for MetS vs. six for pre-frailty), could lead to result in an imbalance in the clustering process, thereby favoring the syndrome with the greater number of variables. To address this, two potential strategies were considered: selecting a subset of variables to limit redundancy or aggregating highly correlated variables by creating synthetic scores or new components derived from a Principal Component Analysis (PCA).

First, an exploratory analysis was conducted, combining qualitative and quantitative approaches. The qualitative approach involved assessing relationships between binary variables through Multiple Correspondence Analysis (MCA) with and contingency table analysis. Concurrently, the quantitative approach centered on the analysis of correlations between continuous variables using a correlation matrix and PCA. This dual approach enabled the evaluation of the consistency of the results and the determination of the most appropriate variable type (binary, continuous, or a combination of both) for HAC. PCA and MCA were performed using R software (version 4.3.3; R Foundation for Statistical Computing, Vienna, Austria). Details of the selection process can be found in Supplementary 4.

The ultimate objective of this process was to achieve a balanced selection of variables for HAC, optimizing the identification of sub-phenotypes while avoiding bias due to the overrepresentation of specific variables or excessive correlations.

##### 2.4.1.3 Sub-phenotypes' characterization

Initially, HAC was performed on the entire study population, and the optimal number of clusters was determined. However, given that the definitions of both MetS and pre-frailty incorporate sex-specific clinical thresholds, it was considered as relevant to assess whether sex played a major role in cluster formation. To this end, dendrograms were generated for the overall population and separately for each sex, to evaluate potential differences in the clustering structure.

To compare the consistency of sex-specific sub-phenotypes with those identified in the overall population, the obtained classes were matched and a contingency table created. This enabled the determination of whether sex-specific sub-phenotypes were fully nested within the globally identified sub-phenotypes or if they exhibited distinct patterns. This evaluation provided insight into whether the analysis should focus solely on global sub-phenotypes or whether a sex-dependent nuances needed to be considered.

To characterize the sub-phenotypes identified through the HAC, the Test-value method was used. This approach identifies the most discriminant clinical criteria by comparing the values observed within each cluster to those expected in the overall population. A significant high absolute test value indicates that a criterion plays a key role in distinguishing the sub-phenotype.

To further describe and understand differences between clusters, chi-square tests were used for categorical variables, while ANOVA or Kruskal–Wallis tests were applied for continuous variables when appropriate. This approach provided a comprehensive assessment of inter-cluster differences, highlighting key clinical, sociodemographic, and lifestyle characteristics of each sub-phenotype.

#### 2.4.2 Factors associated with the sub-phenotypes

In this study, the potential determinant variables were selected based on a comprehensive review of the literature on socio-demographic, lifestyle, and health determinants associated with MetS and/or pre-frailty (see Supplementary 3 for details). In order to identify factors associated with the sub-phenotypes, a multivariable multinomial logistic regression was performed with a four-category dependent variable, representing sub-phenotype membership. To account for missing data in the multivariable analyses, Multiple Imputation by Chained Equations (MICE) using fully conditional specification was performed, generating 10 imputed datasets. Imputation was conducted on all predictors included in the regression model. Results were pooled across imputed datasets using Rubin's rules (32). Interactions between age and sex were tested but were not statistically significant (*p* = 0.44). The following variables were included in the adjusted model: those exhibiting *p* < 0.2 in the bivariate analyses. The linearity assumption for continuous independent variables was assessed using the restricted cubic spline method (33). Model 0 was adjusted for socio-demographic characteristics, Model 1 was further adjusted for lifestyle factors, and Model 2 additionally accounted for health status. When both syndromes shared a common determinant, further analyses were conducted to explore potential differences in its impact. All statistical tests were two-sided, with *p* ≤ 0.05 considered for statistical significance. The analyses were performed using STATA® 18.0.

## 3 Results

Among the 3,398 participants, 73.6% were male and the mean age was 71.9 years (Standard Deviation, SD = 5.5). The overall prevalence of MetS was 44.8%, with 16 distinct observed clinical phenotypes. Pre-frailty affected 47.3% of the sample, with 14 distinct identified clinical phenotypes. Co-occurrence of MetS and pre-frailty was present in 23.2% of participants, with 104 distinct recorded co-occurrent phenotypes (See details Supplementary 7).

### 3.1 Sub-phenotypes' clustering

**Figure 1:**
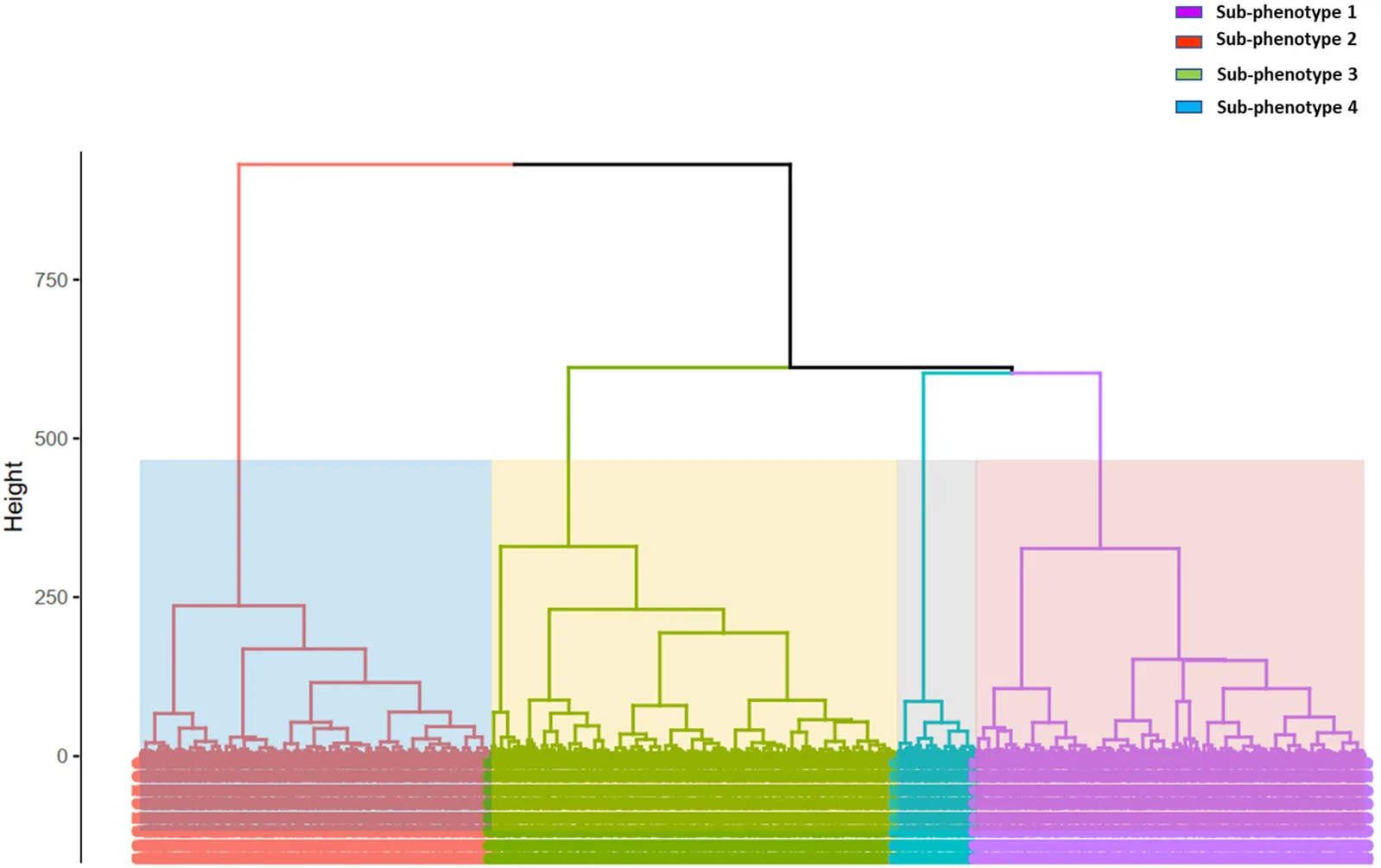
Cluster dendrogram of the overall sample: the dendrogram was constructed using 10 selected variables, including five related to metabolic syndrome (MetS): waist circumference (continuous), fasting blood glucose levels (continuous), triglycerides (TG, continuous), HDL-cholesterol (binary, based on established thresholds), and blood pressure (binary, based on established thresholds); and five related to (pre-)frailty: walking speed (continuous), body weight change (continuous), handgrip strength (continuous), physical activity assessed by energy expenditure (continuous), and exhaustion, categorized as an ordinal variable reflecting different levels of fatigue. Details of the variable selection process are summarized in Supplementary Material 4.

After the selection process, we ended up with 10 variables (5 for MetS and 5 for pre-frailty). Inspection of the dendrogram (Figure 1) supported a 4-cluster structure, which was further corroborated by quantitative internal validation metrics (see details Supplementary 8). Specifically, the Calinski-Harabasz index reached a distinct global maximum at k = 4, and the total within-cluster sum of squares exhibited a clear elbow at k = 4, while average silhouette widths remained comparable between k = 4 and k = 5. Based on this quantitative consensus and clinical parsimony, a four-cluster solution was selected. The distribution of individuals across these clusters was as follows: 31.7% (*n* = 1,078) in sub-phenotype 1 (SP1), 28.7% (*n* = 974) in sub-phenotype 2 (SP2), 6.4% (*n* = 219) in sub-phenotype 3 (SP3), and 33.2% (*n* = 1,127) in sub-phenotype 4 (SP4). Bootstrap resampling validated the internal robustness of the 4-cluster taxonomy. SP3 exhibited exceptional stability (Jaccard index = 0.99), and SP1 showed high stability (Jaccard index = 0.82). SP4 demonstrated acceptable stability (Jaccard index = 0.61). SP2 yielded a Jaccard index of 0.52, reflecting the less stable cluster. The HAC analyses conducted for men and women separately (see Supplementary 5), revealed four groups of similar size to those obtained in the overall population. From the contingency table (data not shown), results showed that among men, 87.5% were classified within the same cluster as in the general analysis, with a discordance of 12.5%. Among women, the concordance was even higher (91.1%), with only 8.9% of discordance. These findings suggest that the overall structure of the sub-phenotypes was largely preserved irrespective of sex, indicating that observed differences were minimal. Consequently, cluster characterization was conducted using the entire population, under the assumption that sex-specific variations were not substantial enough to perform separate analyses.

### 3.2 Characterization of the sub-phenotypes' clusters

#### 3.2.1 Subject reclassification

**Figure 2:**
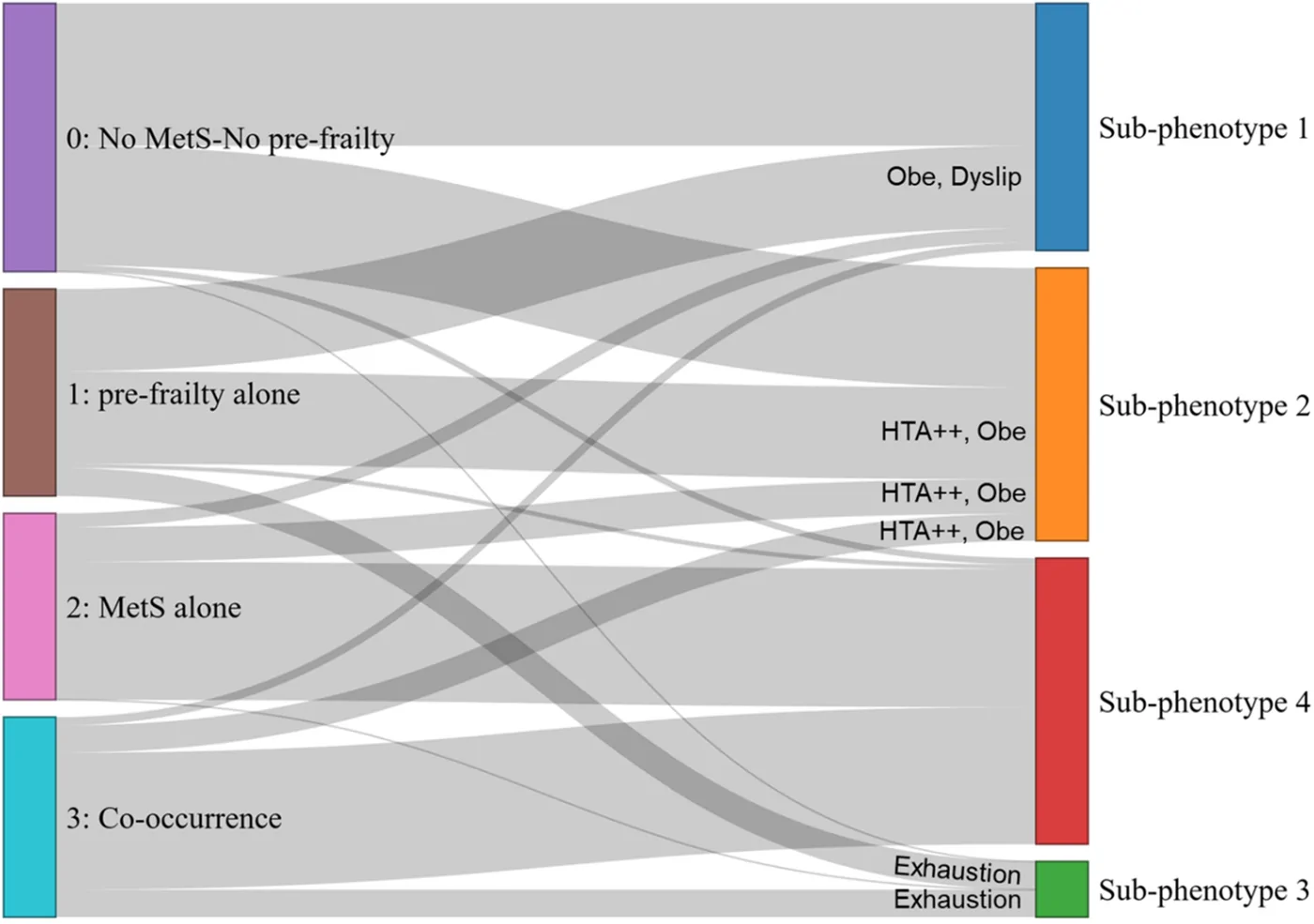
Reclassification of participants from initial MetS and pre-frailty status groups into data-driven sub-phenotypes (Sankey diagram): Participants were initially classified into four groups (no MetS/no pre-frailty, pre-frailty alone, MetS alone, and co-occurrence) and subsequently reassigned into four data-driven sub-phenotypes derived from hierarchical clustering. The labels on the flows (e.g., HTA++, Ob, Dyslip) indicate the presence of the corresponding criteria according to standard diagnostic thresholds (5, 12). Flow thickness is proportional to the number of individuals. HTA, hypertension; Obe, obesity; Dyslip, dyslipidemia.

Clustering analyses based on MetS and pre-frailty criteria, identified four distinct sub-phenotypes. Figure 2 illustrates how subjects from the initial clinical groups (Control, MetS alone, Pre-frailty alone, and MetS–pre-frailty co-occurrence) were redistributed across these clusters. The pre-frailty alone group appeared the most heterogeneous, with 44.5%, 39.7%, and 13.5% of subjects reassigned to SP2, SP1 and SP3, respectively. SP2 was mainly characterized by the presence of hypertension and obesity, as defined by MetS thresholds, whereas all individuals in SP3 presented exhaustion, according to Fried's criteria. MetS alone subjects clustered in SP2 were similarly characterized by hypertension and obesity. Subjects with MetS–pre-frailty co-occurrence were predominantly reassigned to SP4 (68.5%), with smaller proportions reassigned to SP3 (13.7%, also characterized by exhaustion) and SP2 (13.6%, with hypertension and obesity). Finally, among those initially classified as controls, 53.1% (including all those without any positive criteria) clustered in SP1, while 44.6% (with those showing more hypertension and obesity at baseline) were reassigned to SP2.

#### 3.2.2 Description and characterization of the sub-phenotypes

To characterize each sub-phenotype, first, test-values and their associated *p*-values are computed for the 10 variables used in the hierarchical clustering analysis (Table 1), highlighting the overrepresented profiles in the cluster. The sub-phenotypes are then further characterized in terms of clinical and physical parameters, as well as socio-demographic, lifestyle, and psychosocial features, compared with SP1, as a reference group. Clinical and physical parameters and their associated *p*-values (after ANOVA or Kruskal–Wallis) are summarized in Table 2, while the socio-demographic, lifestyle, and psychosocial characteristics are presented in Table 3. Following this workflow, the next section presents the description and characteristics of the 4 clinical sub-phenotypes:

**Table 3:** Characteristics of the study population according to the sub-phenotypes to which the subjects are assigned in the whitehall II cohort study (*n* = 3,398).

Characteristics	Sub-phenotype 1	Sub-phenotype 2	Sub-phenotype 3	Sub-phenotype 4	*p*-value^a^
N (%)	974 (28.7)	1,078 (31.7)	219 (6.4)	1,127 (33.2)	
Age (years, SD)	70.3 (4.8)	72.2 (5.6)	73.04 (5.9)	72.6 (5.5)	**<0**.**0001**
Men (%)	71.8	70.1	64.4	80.3	**<0**.**0001**
Caucasian^b^	95.2	94.1	93.6	91.8	**0**.**01**
BMI (kg/m^2^, SD)	24.7 (3.4)	26.9 (4.3)	27.1 (4.7)	27.9 (4.3)	**<0**.**0001**
BMI categories (%)					**<0**.**0001**
<25	54.8	36.9	32.0	25.5	
25–29.9	37.4	42.6	44.3	49.8	
≥30	7.8	20.5	23.7	24.7	
Marital status (%)					**<0**.**0001**
Single	13.2	12.6	17.8	8.8	
Married/cohabitant	74.1	74.4	59.8	76.6	
Widow/divorced	12.4	12.7	21.0	14.3	
Missing	0.3	0.3	1.4	0.3	
Smoking status (%)					**0**.**02**
Non-smoker	49.8	47.1	44.3	43.0	
Former smoker	43.0	45.7	47.5	50.1	
Current smoker	3.0	2.1	3.6	2.1	
Missing	4.2	5.1	4.6	4.8	
Sedentariness^c^ (%)					**0**.**1**
High	29.8	33.3	32.9	34.5	
Moderate	27.6	28.3	29.2	28.5	
Low	39.6	36.0	32.4	34.2	
Missing	3.0	2.4	5.5	2.8	
Physical activity level (%)					**<0**.**0001**
Less than recommendation	41.5	41.5	53.9	52.7	
Recommendation	58.5	58.5	46.1	47.3	
Alcohol (%)					**0**.**001**
No alcohol in the previous week	16.2	20.5	24.2	21.7	
Moderate, 1–14 units/week	62.9	56.1	58.9	53.9	
High, >14 units/week	20.0	22.4	16.0	22.5	
Missing	0.9	1.0	0.9	1.9	
Fruits and vegetables					**<0**.**0001**
Less than one daily	14.0	18.9	21.9	21.1	
At least once daily	85.4	80.7	78.1	78.5	
Missing	0.6	0.4	0.0	0.4	
Diabetes (%)	2.4	4.1	8.7	17.5	**<0**.**0001**
MetS component (%)					
Hyperglycemia	13.7	22.3	27.4	37.6	**<0**.**0001**
Hypertension	0	93.6	68.0	92.0	**<0**.**0001**
Abdominal obesity	25.1	45.1	51.6	51.8	**<0**.**0001**
Hypertriglyceridemia	28.3	17.9	51.1	97.6	**<0**.**0001**
Low HDL cholesterol	22.7	6.3	48.4	96.8	**<0**.**0001**
Pre-Frailty component (%)					
Shrinking	1.7	3.4	2.7	2.2	0.08
Low handgrip	18.7	22.4	14.2	26.3	**<0**.**0001**
Low physical activity	22.9	24.5	28.3	31.5	**<0**.**0001**
Low gait speed	00.0	4.7	2.3	1.8	**<0**.**0001**
Exhaustion	0.0	0.10	100.0	0.09	**<0**.**0001**
SF36 physical component Score^c^ (%)					**<0**.**0001**
High	42.2	33.5	22.4	28.7	
Medium	33.6	31.3	16.9	34.9	
Low	23.1	32.6	58.9	34.5	
Missing	1.1	2.6	1.8	1.9	
SF36 Mental component Score^c^ (%)					**<0**.**0001**
High	31.5	36.7	10.5	36.3	
Medium	36.2	32.7	17.8	32.1	
Low	31.2	28.0	69.9	29.7	
Missing	1.1	2.6	1.8	1.9	
CESD score^c^					**<0**.**0001**
Case (n, %)	1.5	2.5	29.2	2.6	
Non-case (n, %)	96.2	95.4	66.7	94.2	
Missing	2.3	2.1	4.1	3.2	
GHQ score (%)					**<0**.**0001**
No symptoms	65.3	63.8	22.8	61.0	
At least one symptom	34.3	35.5	75.4	38.1	
Missing	0.4	0.7	1.8	0.9	

Values are sample size (percentages) or mean (SD).

a Analysis of variance or *χ*2 where appropriate;.

b the other group were non-Caucasian;.

c Cut-off: sedentariness (high, moderate, low, this is the cut-off in tertiles of the total time in minutes (2,100, 2,820) spent sitting during the week and at weekends); SF36 physical component score (high, moderate, low, this is the cut-off in tertiles (47.6, 53.3) of the raw score); SF36 mental component score (high, moderate, low, this is the cut-off in tertiles (54.0, 58.3) of the raw score); self-assessment of depression (<20, ≥20, evaluated by the CESD, the threshold of 20 was used to detect people at risk of depression).

First, SP1 was characterized by test-values highlighting an underrepresentation of individuals with hypertension and low HDL-cholesterol, together with lower average triglyceride and fasting glucose levels, and markedly reduced waist circumference compared with the overall sample. Regarding pre-frailty, SP1 was also discriminated by slightly lower handgrip strength and physical activity, but relatively better functional performance with faster walking speed and fewer cases of exhaustion relative to other SPs. Overall, SP1 exhibited comparatively the most favorable cardiometabolic and functional profiles, and also displayed the most advantageous socio-demographic, lifestyle, and psychosocial characteristics within the sample, including younger age, a more balanced sex distribution, lower body mass index (BMI), fewer widowed or divorced individuals, healthier dietary patterns, lower prevalence of smoking and excessive alcohol intake, and higher mental health and quality of life scores. Based on these characteristics, SP1 was selected as the “relatively healthy reference Sub-phenotype” for subsequent comparisons.

Regarding SP2, test-values highlighted an overrepresentation of individuals with hypertension and an underrepresentation of those with low HDL-cholesterol, along with lower average triglyceride and fasting glucose levels compared with the overall sample. For pre-frailty, SP2 showed higher handgrip strength and physical activity, faster gait speed, and fewer cases of exhaustion relative to other SPs. However, compared with SP1, SP2 exhibited less favorable cardiometabolic profile, including higher body weight, waist circumference, blood pressure, and glucose levels. In addition to these clinical differences, SP2 showed distinct socio-demographic structure, being characterized by older age, a higher proportion of women, and more widowed or divorced individuals. Lifestyle and psychosocial features also contrasted from those observed in SP1, including lower fruit and vegetable intake, a higher proportion of former smokers, greater alcohol consumption, and a higher prevalence of depressive symptoms and poor physical quality of life. Overall, SP2 represented a distinct phenotype combining preserved functional characteristics with greater cardiometabolic burden and less favorable lifestyle and psychosocial profiles compared with the reference phenotype. SP2 can be labelled “hypertensive-obese with preserved function Sub-phenotype”.

SP3 was only characterized by pre-frailty components without any of the MetS criteria. Indeed, it included an overrepresentation of individuals with exhaustion, along with lower average physical activity and slower gait speed compared the overall sample. Relative to SP1, SP3 displayed comparatively lower functional performance, including longer walking time, lower energy expenditure. Although MetS variables did not contribute to the clustering, participants in SP3 nonetheless showed metabolic parameters that were less favorable than those in SP1, including higher weight, waist circumference, blood pressure, fasting blood glucose, and triglycerides levels, along with lower HDL-cholesterol. Beyond clinical and functional measures, SP3 also differed from SP1 in socio-demographic and lifestyle characteristics, being composed of older individuals, a higher proportion of women, and more widowed, divorced, or single participants. Lifestyle behaviors were comparatively less favorable, with more former smokers, lower physical activity, and lower fruit and vegetable consumption, although fewer individuals exceeded recommended alcohol intake. Psychosocial outcomes were also relatively poorer, with higher rates of depressive symptoms, psychological distress (GHQ), and low scores on both physical and mental components of the SF-36. Overall, SP3 was characterized by predominant pre-frailty features alongside functional, metabolic, socio-demographic, lifestyle, and psychosocial characteristics that were less favorable relative to SP1. SP3 can be labelled the “psychosocial vulnerability Sub-phenotype”.

Finally, SP4 was characterized by test-values showing an overrepresentation of individuals with hypertension and low HDL-cholesterol, along with higher mean waist circumference, triglycerides, and fasting glucose compared with the overall sample. For pre-frailty components, this cluster also showed lower average handgrip strength and physical activity, slower gait speed, and an overrepresentation of individuals reporting exhaustion. Compared with SP1, SP4 exhibited less favorable clinical measures, including higher weight, waist circumference, blood pressure, fasting glucose, and triglycerides levels, as well as lower concentrations of HDL-cholesterol. Functional capacity was also relatively lower, with longer walking time and reduced physical activity. SP4 further included a higher prevalence of type 2 diabetes. Beyond clinical and functional measures, SP4 differed from SP1 in socio-demographic and lifestyle characteristics with older age, a higher proportion of men, more widowed or divorced individuals, and higher BMI. Lifestyle behaviors were comparatively less favorable, including more former smokers, greater alcohol consumption, lower physical activity, and fruit and vegetable intake. Psychosocial indicators were also relatively poorer, with more depressive symptoms, psychological distress (GHQ), and lower scores on both the physical and mental components of the SF-36. Overall, SP4 was characterized by comparatively less favorable metabolic, functional, socio-demographic, lifestyle, and psychosocial characteristics relative to SP1. SP4 can be labelled combined “metabolic and functional impairment Sub-phenotype”

### 3.3 Risk factors associated with sub-phenotypes

Table 4 presents the odds ratios for the likelihood of belonging to SP2, SP3, and SP4 compared to the reference group (SP1). These data are based on a multivariable multinomial logistic regression model adjusted for socio-demographic, lifestyle, and health-related factors. Utilizing SP1 as a reference thus facilitated the assessment of the factors associated with deviations from the most protective healthy profile. The model displayed in Table 4 corresponds to the primary and most comprehensive model (Model 3), incorporating all covariates. Models with more limited adjustment, Model 1 (socio-demographic characteristics only) and Model 2 (socio-demographic and lifestyle variables), are presented in Supplementary 6.

**Table 4:** Multivariable associations (OR and 95% CI using multinomial logistic regression model) showing the relationships between socio-demographic, lifestyle and health status characteristics and sub-phenotypes groups membership.

Characteristics	Sub-phenotype 2		Sub-phenotype 3		Sub-phenotype 4		*p*-value
	OR (95% CI)	*p*-value	OR (95% CI)	*p*-value	OR (95% CI)	*p*-value	
Age, years	1.07 [1.05; 1.08]	<0.0001*	1.09 [1.06; 1.12]	<0.0001*	1.07 [1.06; 1.09]	<0.0001*	<0.0001*
Sex							<0.0001*
Men	0.84 [0.68; 1.04]		0.85 [0.58; 1.23]		1.56 [1.25; 1.96]		
Women	1 (ref)	0.1	1 (ref)	0.3	1 (ref)	<0.0001*	
Ethnicity							0.09
Caucasian	1 (ref)	0.8	1 (ref)	0.5	1 (ref)	0.06	
Non-Caucasian	1.03 [0.69; 1.55]		0.81 [0.40; 1.62]		1.44 [0.98; 2.13]		
Marital status							0.07
Single	0.87 [0.66; 1.15]	0.3	1.10 [0.69; 1.75]	0.7	0.69 [0.51; 0.93]	0.01	
Married/cohabitant	1 (ref)	0.3	1 (ref)	0.7	1 (ref)	0.04	
Widow/divorced	0.84 [0.63; 1.12]	0.2	1.20 [0.76; 1.88]	0.4	1.03 [0.78; 1.36]	0.8	
Alcohol consumption							0.006*
No in the previous week	1 (ref)	0.007*	1 (ref)	0.9	1 (ref)	0.001*	
Moderate, 1–14 units/wk	0.75 [0.59; 0.96]	0.02*	0.91 [0.60; 1.40]	0.6	0.66 [0.52; 0.84]	0.001*	
High, >14 units/wk	1.02 [0.76; 1.38]	0.8	0.99 [0.52; 1.58]	0.7	0.83 [0.62; 1.12]	0.22	
Fruits and vegetables intakes							
Less than one daily	1.47 [1.15; 1.88]		1.45 [0.96; 2.18]		1.59 [1.25; 2.02]		0.002*
At least once daily	1 (ref)	0.002*	1 (ref)	0.07*	1 (ref)	<0.0001*	
Smoking status							0.36
Non-smoker	1 (ref)	0.2	1 (ref)	0.5	1 (ref)	0.07	
Former smoker	1.11 [0.92; 1.33]	0.2	1.19 [0.85; 1.66]	0.3	1.22 [1.01; 1.46]	0.03	
Current smoker	0.75 [0.42; 1.33]	0.3	1.21 [0.48; 3.03]	0.6	0.84 [0.48; 1.47]	0.5	
Sedentarily behavior							0.18
High	1.18 [0.96; 1.46]	0.1	1.01 [0.69; 1.50]	0.9	1.35 [1.09; 1.67]	0.006	
Moderate	1.07 [0.86; 1.33]	0.5	1.12 [0.75; 1.68]	0.5	1.19 [0.96; 1.49]	0.1	
Low	1 (ref)	0.3	1 (ref)	0.8	1 (ref)	0.02	
SF36 physical component Score							
High	1 (ref)	0.02*	1 (ref)	<0.0001*	1 (ref)	0.0001*	
Medium	1.06 [0.86; 1.32]	0.5	0.98 [0.67; 1.58]	0.9	1.38 [1.11; 1.71]	0.003*	
Low	1.38 [1.09; 1.75]	0.007*	3.06 [2.03; 4.61]	<0.0001*	1.65 [1.30; 2.10]	<0.0001*	
SF36 Mental component Score							
High	1 (ref)	0.3	1 (ref)	<0.0001*	1 (ref)	0.4	
Medium	0.90 [0.72; 1.11]	0.3	1.69 [0.98; 2.92]	0.06	0.87 [0.70; 1.10]	0.2	
Low	0.83 [0.65; 1.06]	0.1	3.32 [1.99; 5.55]	<0.0001*	0.88 [0.69; 1.12]	0.3	
CESD score							<0.0001*
Case	1.49 [0.81; 2.73]		3.10 [1.94; 4.96]		1.37 [0.74; 2.55]		
Non-case	1 (ref)	0.20	1 (ref)	<0.0001*	1 (ref)	0.31	
GHQ score							0.0002*
No symptom	1 (ref)	0.39	1 (ref)	<0.0001*	1 (ref)	0.98	
At least one symptom	1.26 [0.74; 2.17]		2.31 [1.55; 3.45]		1.00 [0.63; 1.60]		

Odds ratios for the likelihood of belonging to sub-phenotypes 1, 3, and 4 compared to the reference group (sub-phenotype 1). Characteristics are adjusted on each other.

* Significative *P*-value.

Membership in SP2 was primarily driven by advancing age and lifestyle factors. Older age increased the likelihood of belonging to SP2 (OR: 1.07 per year [1.05–1.08]), while moderate alcohol consumption was inversely associated compared with abstinence (OR: 0.75 [0.59–0.96]). Low fruit and vegetable intake and poor physical quality-of-life were each independently associated with SP2 membership (OR: 1.47 [1.15–1.88] and OR: 1.38 [1.09–1.75], respectively).

SP3 membership was dominated by psychosocial and mental health determinants. Beyond advancing age (OR: 1.09 [1.06–1.12]), depressive symptoms (OR: 3.10 [1.94–4.96]) and psychological distress (OR: 2.31 [1.55–3.45]) were the strongest independent predictors. Poor physical and mental quality-of-life further markedly increased the likelihood of belonging to this sub-phenotype (OR: 3.06 [2.03–4.61] and OR: 3.32 [1.99–5.55], respectively), highlighting the predominant role of mental vulnerability in this profile.

SP4 membership was primarily driven by demographic and lifestyle factors. Beyond advancing age (OR: 1.07 [1.06–1.09]), male sex was associated with a higher likelihood of belonging to this sub-phenotype (OR: 1.56 [1.25–1.96]). Moderate alcohol consumption was inversely associated with SP4 membership (OR: 0.66 [0.52–0.84]), whereas low fruit and vegetable intake (OR: 1.59 [1.25–2.02]) and both low and medium physical SF-36 component scores (OR: 1.65 [1.30–2.10] and OR: 1.38 [1.11–1.71], respectively) independently increased the likelihood of belonging to this group.

## 4 Discussion

The present study aimed to identify clinical sub-phenotypes by jointly considering the dimensions of MetS, as defined by the harmonized definition, and pre-frailty, according to the Fried physical frailty phenotype. Using an unsupervised hierarchical clustering approach, four distinct sub-phenotypes were identified, relative to different health conditions. The present work operates at the clinical and epidemiological level, jointly integrating the defining criteria of both MetS and pre-frailty. In addition, sociodemographic, lifestyle and psychosocial factors were significantly associated with sub-phenotype membership, underscoring the multidimensional nature of vulnerability in older adults.

The stratification of complex clinical states into sub-phenotypes has emerged as a promising approach in the context of personalized medicine. Traditional binary diagnostic classifications may obscure heterogeneity among individuals who meet the same diagnostic label, limiting both risk prediction and tailored intervention (34–36). Conditions such as MetS and obesity encompass diverse biological and clinical presentations, and prior studies have shown that identifying “metabolically healthy” vs. “unhealthy” obesity (37, 38) clarifies heterogeneity in cardiometabolic risk (38, 39). Importantly, these profiles are dynamic, with individuals transitioning from lower- to higher-risk states over time. This dynamic nature echoes findings from studies on other chronic conditions, including type 2 diabetes (36), hepatic steatosis (40), masked hypertension (41), where unsupervised clustering revealed clinically distinct subgroups with differential prognosis. Our results extend this approach to the joint domain of MetS and pre-frailty, providing a more nuanced characterization of older adults and a structure of the heterogeneity of this complex co-occurrence.

SP1 represented the most favorable profile, with good metabolic parameters and preserved functional capacity, consistent with the concept of “resilient agers”. Such resilience phenotypes have been reported across cohorts and are consistently associated with reduced morbidity, disability and mortality, suggesting a trajectory of healthy aging (42–44). By contrast, SP4 was characterized by co-occurring metabolic and functional alterations, including reduced physical performance in relation to pre-frailty. Although mean values of some parameters did not exceed diagnostic thresholds, their co-occurrence indicated a state of possible heightened vulnerability (18). Previous studies highlighted the role of frailty in mediating the relationships between MetS and mortality (18), while concepts such as dynapenic obesity (the combination of abdominal obesity and low muscle strength) have been linked to greater risk of falls (45), mortality, and may also act as a precursor to both MetS (46) and frailty (47). The co-occurrence of MetS parameter and frailty functional alterations was shown more prevalent in aging and may be driven by shared underlying mechanisms such as low-grade chronic inflammation, insulin resistance, and oxidative stress, which are recognized as common pathophysiological pathways of both MetS and frailty (11, 48). SP3 was primarily distinguished by functional and psychosocial features rather than metabolic parameter. Mechanistically, this pattern may reflect an underlying imbalance in energy metabolism, possibly related to mitochondrial dysfunction, low-grade chronic inflammation, or neuroendocrine dysregulation (49, 50). Such factors may impair motivation, increase perceived exertion, and negatively affect daily functioning. The integration of psychosocial factors further suggests that conventional frailty definitions, focused predominantly on physical indicators, may fail to capture atypical yet clinically meaningful profiles of vulnerability. Finally, SP2 showed relatively preserved metabolic and functional characteristics but a higher prevalence of hypertension compared with SP1. This cluster may represent a group with early or partial metabolic alterations, Comparable to “pre-MetS” or intermediate phenotypes that were described in other studies, where individuals with one or two MetS criteria exhibited elevated risks of type 2 diabetes or cardiovascular diseases (51) despite falling below diagnostic thresholds.

Regarding the internal validity and stability of the observed sub-phenotypes, bootstrap resampling offered further support for the robustness and reproducibility of the proposed taxonomy. The stability observed for SP1 and SP4 suggests that both the healthy reference profile and the co-occurring functional and metabolic disturbance group represent relatively coherent clinical patterns. Notably, the high stability of SP3 suggests that isolated functional and psychosocial patterns may represent a reproducible clinical phenotype rather than being solely driven by sampling variability. In contrast, the moderate stability of SP2 is consistent with an intermediate clinical spectrum (isolated hypertension with preserved metabolic and functional capacities) that partially overlaps with neighboring clusters rather than forming a strictly isolated subgroup. In population-based studies of older adults, partially overlapping clinical profiles are expected because cardiometabolic risk accumulates along a continuum rather than through discrete disease states, making intermediate phenotypes clinically plausible (52, 53).

The present findings highlight that not all the criteria used to define MetS or pre-frailty contribute equally to the structure of the identified sub-phenotypes. For instance, although waist circumference is a core component of MetS definition, it did not significantly discriminate SP2. Similarly, despite being included in the Fried criteria for pre-frailty, weight variation did not emerge as a key differentiator for SP3, which nonetheless exhibited marked functional impairment. These observations suggest that the presence or absence of a single criterion may not be sufficient to define a clinically meaningful profile. Instead, it is the specific combination of features and their interactions across multiple physiological components that gives the sub-phenotypes their coherence and relevance. This raises concerns about approaches that rely exclusively on binary thresholds or additive scoring systems, as these may generate artificial clinical phenotypes that fail to reflect the true clinical and pathophysiological complexity and continuum. In contrast, unsupervised methods allow empirically grounded profiles to emerge that better capture and structure population heterogeneity. These methods are aligned with the principles of personalized medicine, offering a more nuanced understanding of health status in aging populations. They may also lay the groundwork for targeted stratification using molecular profiling techniques, such as metabolomics, as demonstrated by Pujos-Guillot et al. (7) to facilitate earlier and more precise preventive strategies.

Among the factors associated with the identified sub-phenotypes, age and a low physical component score on the SF-36, were common factors across the three sub-phenotypes, consistent with their known established association with metabolic and functional decline in aging (54–57). SP2 and SP4 shared lifestyle-related factors, with moderate alcohol consumption and low fruit and vegetable intake, both associated with these sub-phenotypes, consistent with established links between these lifestyle factors and healthy aging (58, 59). Some factors appeared to be more specific: male sex was uniquely associated with SP4, which aligns with epidemiological studies showing a higher prevalence of metabolic abnormalities in aging men (60). In contrast, SP3 was strongly associated with psychological and mental health-related variables, including depressive symptoms, psychological distress, and a low mental component score on the SF-36. These findings highlight the relevance of mental health vulnerability as an important dimension associated with functional frailty profiles, a dimension still often underestimated in biomedical models focused solely on physical health (61, 62).

This study has several limitations that should be acknowledged. First, unsupervised clustering involves subjective methodological choices, such as the selection of the number of clusters retained and distance metrics. In this study, the number of clusters was guided by visual inspection of the dendrogram and supported by objective sensitivity analyses, which confirmed the stability of the clustering structure. Such an approach is acceptable in exploratory analyses and facilitated the identification of sub-phenotypes that are interpretable and clinically meaningful. Second, the cross-sectional design precludes causal inference and does not allow assessment of temporal transitions between phenotypes. Third, the use of binary representations for some MetS and frailty criteria may have reduced the ability to capture within-criterion variability and potentially influenced cluster boundaries. Although this approach was chosen to preserve clinically relevant threshold-based definitions, including treatment status where applicable, it may have masked differences in the severity of certain abnormalities. Fourth, some variables (notably exhaustion and depression) were self-reported and may be subject to reporting bias. Firth, The association between SP3 and poor mental health should be interpreted considering the potential overlap between exhaustion, a component of the Fried phenotype, and depressive symptoms, as the exhaustion criterion is derived from CES-D items. Future studies using more specific mental health assessments may help further disentangle these relationships. Finally, the relatively homogeneous European population, predominantly composed of older male participants from an occupational cohort, may limit the generalizability of these findings to more diverse settings, including populations with different ethnic, socioeconomic, or occupational backgrounds. Validation of the identified sub-phenotype structure in independent and more diverse cohorts will be required to assess its reproducibility and broader applicability.

The strengths of this study include its large sample size, the joint assessment of two syndromes that are rarely studied together, and the use of an unsupervised clustering method enabling the empirical identification of clinically coherent sub-phenotypes. In addition, stratified analyses showed consistent patterns across sexes, lending robustness to our classification.

Building on these strengths, this study offers several promising research and clinical perspectives. Future research should validate these sub-phenotypes in independent cohorts and assess their longitudinal stability to better understand their clinical significance. Follow-up analyses could determine whether these profiles predict incident disability, cardiovascular events, hospitalization, or mortality. Integrating biological markers, such as inflammatory cytokines, metabolomics, and genetic data may provide mechanistic insights and biomarkers for early detection. Interventional studies are also warranted to test whether phenotype-specific strategies can alter trajectories and improve outcomes.

In conclusion, this study identified four distinct sub-phenotypes defined by the joint expression of MetS and pre-frailty, ranging from more favorable clinical profiles to profiles characterized by greater metabolic or psychosocial burden. These findings highlight the heterogeneity of aging-related health profiles and support the value of multidimensional approaches for characterizing complex geriatric conditions. By combining metabolic, functional, and psychosocial domains, sub-phenotyping provides a promising framework for improving the characterization of older adults' health profiles and generating hypotheses for future precision geriatric research and targeted health promotion strategies.

## Data Availability

Publicly available datasets were analyzed in this study. This data can be found here: Data, protocols, and other metadata of the Whitehall II study are available for sharing within the scientific community. Bona fide researchers interested in accessing the data can apply through the Dementias Platform UK (https://www.dementiasplatform.uk) or the Whitehall Scientific Committee (https://www.ucl.ac.uk/psychiatry/research/mental-health-older-people/whitehall-ii/data-sharing).
