# Supplementary material for: Clinical sub-phenotypes of co-occurring metabolic syndrome and pre-frailty and their associated factors in older adults: findings from Whitehall II study
Source: Front Med (Lausanne). 2026 Sep 8;13:1909752. doi: 10.3389/fmed.2026.1909752 (PMC13593485; doi:10.3389/fmed.2026.1909752)
Supplement: Supplementary file 1 [file Supplementaryfile1.docx]

**Supplemental materials**

Table of contents

[Supplemental 1: Metabolic syndrome and Pre-frailty definition	1](#_Toc236961855)

[Supplemental 2: Flowchart of the subject selection	3](#_Toc236961856)

[Supplemental 4: Results of variable selection	4](#_Toc236961857)

[Supplemental 5: Sex specific cluster dendrograms	12](#_Toc236961858)

[Supplemental 6: Multivariable associations with limited covariable adjustment.	14](#_Toc236961859)

[Supplemental 7: Distribution of clinical combination of MetS, Pre-frailty and MetS-Pre-frailty co-occurrence	18](#_Toc236961860)

[Supplemental 8: Internal validation metrics for optimal cluster selection (k = 2 to 10).	22](#_Toc236961861)

# Supplemental 1: Metabolic syndrome and Pre-frailty definition

Metabolic syndrome was defined as the simultaneous presence of three or more of the following criteria, as defined by the consensus threshold(12) :

1) Abdominal obesity (waist circumference ≥102 cm and ≥88 cm, for men and women, respectively);

2) Elevated blood levels of triglycerides (TG) (serum level ≥1.7 mM or the use of lipid-modifying medications);

3) Low blood levels of HDL-Cholesterol (HDL-chol) (<1.0 mM and <1.3 mM for men and women, respectively or the use of lipid-lowering medications);

4) Elevated fasting blood glucose (≥5.6 mM or the use of glucose-lowering medications);

5) Elevated blood pressure (systolic blood pressure (SBP) ≥130 mmHg and/or diastolic blood pressure (DBP) ≥85 mmHg or the use of antihypertensive medications).

*Pre-frailty* was defined as the presence of one or two criteria of the Fried’s phenotype(5), hereafter listed:

1) *Exhaustion*: assessed using two items from the Centre for Epidemiologic Studies- Depression (CES-D) scale (25) : “I felt that everything I did was an effort” or “I could not get going”. Participants who answered “occasionally or moderate amount of the time (3-4 days)” or “most or all of the time (5-7 days)” to either of the item were categorized as exhausted.

2) *Physical activity*: low levels of physical activity were defined as an expenditure of less than 383 and 270 kcal/week for men and women, respectively, calculated in the cohort as previously described(26)

3) *Walking speed*: the result was recalculated according to the established threshold, with participants classified as having a slow walking speed when the time required to walk a distance of 2.4 m was ≥3.73 s (with a height ≤173 cm for men or ≤159 cm for women) or ≥3.20 s (with a height >173 cm for men or >159 cm for women)

4) *Grip strength*: measured using the Smedley hand grip dynamometer. Low grip strength was defined as ≤29 kg (BMI ≤24 kg/m^2^), ≤30 (BMI 24.1–28), ≤32 (BMI >28) for men and ≤17 (BMI ≤23), ≤17.3 (BMI 23.1–26), ≤18 (BMI 26.1–29), ≤21 (BMI >29) for women.

5) *Weight loss*: originally defined as an unintentional loss of ≥5% body weight over the past year(5). In the absence of such data, we used a proxy definition: a ≥10% decrease compared with the weight measured at the previous study phase, as applied in the Women's Health Aging Study I(27).

# Supplemental 2: Flowchart of the subject selection

 Supplemental 3: Assessed determinants of sub-phenotypes

In this study, the potential determinant variables were selected based on a comprehensive review of the literature on socio-demographic, lifestyle, and health determinants associated with MetS and/or pre-frailty. The socio-demographic and anthropometric variables included sex (male, female), ethnicity (Caucasian, non-Caucasian), age (continuous), marital status (single, married/cohabiting, widowed/divorced), and BMI (<25, 25–29.9, ≥30 kg/m²). The lifestyle factors considered were smoking status (non-smoker, former smoker, current smoker), alcohol consumption (no alcohol in the previous week, 1–14 units/week, >14 units/week), fruit and vegetable intake (less than one portion/day, at least one portion/day), sedentary behavior, and physical activity. Sedentary behavior was categorized as high, moderate, or low, based on the tertile distribution of total sitting time during the week and weekends (cut-offs: 2,100 and 2,820 min). Physical activity was categorized as ≤2.5 or >2.5 hrs/week, representing the total time spent in moderate-to-intense activity. The SF-36 physical and mental component scores were used to assess physical and mental health indicators, respectively. These component scores were categorized as low, moderate, or high based on the tertile distribution of their raw scores (physical: 47.6, 53.3; mental: 54.0, 58.3). The presence of depressive symptoms was assessed using the CES-D scale, with a score of ≥20 indicating an elevated risk of depression. Psychological distress and mental health symptoms were evaluated using the General Health Questionnaire (GHQ-30). The classification of type 2 diabetes was determined based on the following criteria: fasting glucose levels (≥7 mM) or the utilization of diabetes medication.

# Supplemental 4: Results of variable selection

The correlation matrix analysis (Suppl. 4 Figure 1) revealed a strong correlation, defined as Pearson’s r > 0.5, between SBP and DBP (r = 0.67), as well as between the two CES-D components assessing exhaustion (r = 0.53). Despite their interdependence, SBP and DBP information was retained separately in the following evaluation due to their distinct clinical relevance. Similarly, the two CES-D components were first kept as two variables in the following evaluation.

**Suppl. 4 Figure 1:** **Correlation matrix analysis**

The exploratory principal component analysis (PCA) of the multivariate relationships among the variables of both syndromes, performed after mean centering and scaling, identified six dimensions of interest explaining a cumulative variance of 64.5%. This selection was based on the Kaiser criterion applied to the scree plot of eigenvalues (Suppl. 4 Figure 2).

**Suppl. 4 Figure 2:** **Scree plot of eigenvalues from the principal component analysis (PCA)**

The principal component analysis reveals distinct yet interconnected dimensions (Suppl. 4 Figs. 3). The Dim1–Dim2 factorial plane highlights some expected relationships between most of the MetS components, with an additional notable illustration of a link between waist circumference and triglyceridaemia. The Dim3–Dim4 plane reveals links between variables characterizing frailty, particularly the strong relationship between CES-D Item1 and CES-D Item2, but also links between Handgrip Strength and Physical Activity, in opposition to the Walking Speed. The Dim5–Dim6 plane emphasizes the relationships between the use of medication and metabolic parameters, as illustrated for example by the similar correlation between glycaemia and the use of glucose-lowering medication.

**A**

**B**

**C**

**Suppl. 4 Figure 3:** **Principal component analysis (PCA) of MetS and (Pre-)frailty variables**. Variable factor maps are shown for the first six principal components. **(A)** Dimension 1–Dimension 2 factorial plane; (B) Dimension 3–Dimension 4 factorial plane; (C) Dimension 5–Dimension 6 factorial plane. Variables were centered and scaled to unit variance prior to PCA. The position of each variable reflects its contribution and correlation structure across the principal component space.

To assess the consistency of these observations, a multiple correspondence analysis (MCA) was conducted on the qualitative variables used to define the syndromes. This approach enables for the examination of whether analogous relationships emerge when variables are categorized, thereby providing additional insight into the robustness of the observed relations.

The MCA was performed on all binary qualitative variables that defined the two syndromes, in accordance with the thresholds, established by Alberti et al.(12) for MetS and by Fried et al.(5) for pre-frailty. This analysis identified three key dimensions, selected based on Kaiser’s criterion, which together explain 44.05% of the cumulative variance (Suppl. 4 Figures 4 & 5). The results showed an overall trend that opposed positive modalities of the criteria, which grouped participants who met the threshold for metabolic disturbances or pre-frailty indicators, to the negative modalities, which grouped those who did not meet these criteria. This structuring was observed for both MetS and pre-frailty criteria. With respect to MetS, which drives the first dimension, a strong concordance is observed between the positive and negative modalities of HDL-chol and TG, indicating a stable relationship between these two metabolic parameters, regardless of their classification. The occurrence of these two criteria appeared to be associated also to the positive modality of glycemia, while the non-occurrence seems linked to negative modality of the blood pressure criterion. For the pre-frailty criteria, the MCA brings to forefront the specificities of the positive modalities of Walking Speed and Exhaustion, as observed in the PCA, but also the Weight Loss, while links between Handgrip Strength and Physical Activity are less noticeable.

Overall, taking into account that the variables used for the MCA are limited compared to the diversity in the ones for PCA, the MCA demonstrates some consistency with the PCA results, while highlighting in a simpler way the individual criteria that have notable specificities. This reinforces the structure previously observed in the PCA and underscores the relevance of the identified variable groups among criteria.

The analysis of the relationship between quantitative and binary variables defining MetS and pre-frailty revealed a general concordance of results on the link among criteria. This finding could suggest that HAC could be performed using either quantitative variables, binary variables (defined by thresholds), or a combination of both, as long as intra-criterium redundancy is handled, even though the quantitative variables may enable more diversity in the studied variability. The final choice of variables was guided by clinical relevance and the choices that maximized the extracted information while preserving adequate data variability.

Indeed, optimizing the integration of these variables into the hierarchical clustering algorithm is necessary to circumvent redundancy while preserving nuances when possible. Since the MetS and pre-frailty revealed only few redundancies between the two groups of variables, the selection of variables to retain for HAC was made independently. Concerning the pre-frailty variables, the choice for the criteria based on only one quantitative variable each, was to keep the corresponding continuous variables. For the Exhaustion criterium, the two CES-D components were aggregated into a single ordinal categorical variable, defining exhaustion in a graded manner (0: not exhausted, 1: moderate exhaustion, 2: severe exhaustion) rather than as a binary classification, in line with Fried’s criteria.

Concerning the MetS variables, additional checks were performed to help the selection. If the Waist Circumference criteria could easily be set to the continuous measure, the four other criteria included several variables. With regard to blood pressure, the binary variable was retained on the basis of predefined thresholds. This decision was justified by the need to maintain clinically relevant information on SBP and DBP while also incorporating data on antihypertensive medication use, that has an impact on the measured quantitative variables (with a notable part of individuals that are clinically labelled as positive for the blood pressure criteria due to medication, but have quantitative SBP and DBP values lower than the two thresholds – data not shown). Concerning the blood glucose criterium, the question of the medication impact was also investigated to help selecting a unique variable for HCA. Among the individuals classified as positive by the blood glucose criterion, only 46 individuals (5.4%) were classified as positive solely on the basis of the medication use. These findings indicate that within the study sample, the contribution of the medication variable to the definition of the blood glucose criterion was minimal. Consequently, it was decided to retain only the continuous blood glucose variable in the HAC, to favor the variability of the data while accepting only a limited loss regarding the medication-related information. Concerning the last two criteria, TG and HDL-chol, an additional difficulty was aroused due to the criteria sharing information in their clinical individual classification, through the lipid-lowering medication use. Exploratory analyses using PCA and MCA revealed a strong link between the two dyslipidaemia parameters, with in particular MCA highlighting a strong co-occurrence regarding the clinical classification of individuals. To objectively assess this relationship and guide methodological decisions, a contingency table was built to evaluate the concordance between the two dyslipidaemia criteria, with details about the role of medication in the clinical classification (data not shown). This analysis identified a positive discrepancy in case classification in 248 individuals, of whom 220 were not taking lipid-lowering medication, highlighting the need for an approach that preserves continuous values. However, the medication used also accounted for an important number of cases with no quantitative values beyond the thresholds, thus this information was also crucial to keep. A methodological compromise was therefore adopted: as the HDL cholesterol case attribution appeared to be mainly driven by the medication use, the binary HDL-chol criterion was retained for HCA, inherently including information on medication use, while the TG variable was retained in its continuous form to preserve greater data variability (without losing the medication information in the overall analysis, as it is already included in the binary HDL-chol criterion). This approach aims at an optimal consideration of the information about dyslipidaemic parameters while avoiding over-representation in the HCA analysis.

In summary, the process of selecting variables for clustering was undertaken with the objective of retaining clinically relevant variables in the HAC while maximizing information and variability without introducing bias due to the over-representation of certain criteria. The variables selected for MetS include waist circumference (continuous), fasting levels of blood glucose (continuous), TG (continuous), and HDL-chol (binary, based on established thresholds), and blood pressure (binary, based on established thresholds). For pre-frailty, the retained variables were: walking speed (continuous), body weight change (continuous), handgrip strength (continuous), physical activity measured by energy expenditure (continuous), and exhaustion, which was categorized as an ordinal variable to represent different levels of fatigue.

**Suppl. 4 Figure 4:** **Dim1 – Dim2 factorial plane MCA**

**Suppl.4 Figure 5:** **Dim1 – Dim3 factorial plane MCA**

# Supplemental 5: Sex specific cluster dendrograms

**Suppl 5. Figure 1:** **Cluster dendrogram from women samples:** The dendrogram was constructed using 10 selected variables, including five related to metabolic syndrome (MetS): waist circumference (continuous), fasting blood glucose levels (continuous), triglycerides (TG, continuous), HDL-cholesterol (binary, based on established thresholds), and blood pressure (binary, based on established thresholds); and five related to (pre-)frailty: walking speed (continuous), body weight change (continuous), handgrip strength (continuous), physical activity assessed by energy expenditure (continuous), and exhaustion, categorized as an ordinal variable reflecting different levels of fatigue. Details of the variable selection process are summarized in Supplementary Material 4.

**Suppl 5. Figure 2:** **Cluster dendrogram from men sample :** The dendrogram was constructed using 10 selected variables, including five related to metabolic syndrome (MetS): waist circumference (continuous), fasting blood glucose levels (continuous), triglycerides (TG, continuous), HDL-cholesterol (binary, based on established thresholds), and blood pressure (binary, based on established thresholds); and five related to (pre-)frailty: walking speed (continuous), body weight change (continuous), handgrip strength (continuous), physical activity assessed by energy expenditure (continuous), and exhaustion, categorized as an ordinal variable reflecting different levels of fatigue. Details of the variable selection process are summarized in Supplementary Material 4.

# Supplemental 6: Multivariable associations with limited covariable adjustment.

**Suppl 6. Table 1: (Model 1) Multivariable associations (OR and 95% CI using multinomial logistic regression model) between socio-demographic characteristics and sub-phenotypes group membership.**

|  |  | **Sub-phenotype 2** | | |  | | **Sub-phenotype 3** | | | |  | | **Sub-phenotype 4** | | | |  | | p-value | |  |
| --- | --- | --- | --- | --- | --- | --- | --- | --- | --- | --- | --- | --- | --- | --- | --- | --- | --- | --- | --- | --- | --- |
|  |  |  |  | |  | |  | |  | |  | |  | |  | |  | |  | |  |
| N=3,398 |  | OR (95% CI) | *p*-value | |  | | OR (95% CI) | | *p*-value | |  | | OR (95% CI) | | *p*-value | |  | |  | |  |
|  |  |  |  | |  | |  | |  | |  | |  | |  | |  | |  | |  |
| Age, years |  | 1.07 [1.05 ; 1.09] | <0.0001 | |  | | 1.09 [1.07 ; 1.12] | | <0.0001 | |  | | 1.08 [1.06 ; 1.10] | | <0.0001 | |  | | <0.0001 | |  |
|  |  |  |  | |  | |  | |  | |  | |  | |  | |  | |  | |  |
| Sex |  |  |  | |  | |  | |  | |  | |  | |  | |  | | <0.0001 | |  |
| Men |  | 0.88 [0.72 ; 1.07] | 0.1 | |  | | 0.81 [0.58 ; 1.13] | | 0.2 | |  | | 1.57 [1.27 ; 1.95] | | <0.0001 | |  | |  | |  |
| Women |  | 1 (ref) |  | |  | | 1 (ref) | |  | |  | | 1 (ref) | |  | |  | |  | |  |
|  |  |  |  | |  | |  | |  | |  | |  | |  | |  | |  | |  |
| Ethnicity |  |  | |  | |  | |  | |  | |  | |  | |  | |  | | 0.01 | |
| Caucasian |  | 1 (ref) |  | |  | | 1 (ref) | |  | |  | | 1 (ref) | |  | |  | |  | |  |
| Non-Caucasian |  | 1.13 [0.76 ; 1.68] | 0.5 | |  | | 1.22 [0.65 ; 2.27] | | 0.5 | |  | | 1.76 [1.21 ; 2.56] | | 0.003 | |  | |  | |  |
|  |  |  |  | |  | |  | |  | |  | |  | |  | |  | |  | |  |
| Marital status |  |  |  | |  | |  | |  | |  | |  | |  | |  | | 0.003 | |  |
| Single |  | 0.93 [0.71 ; 1.22] | 0.6 | |  | | 1.58 [1.04 ; 2.41] | | 0.03 | |  | | 0.76 [0.57 ; 1.01] | | 0.09 | |  | |  | |  |
| Married/cohabitant |  | 1 (ref) | 0.5 | |  | | 1 (ref) | | 0.01 | |  | | 1 (ref) | | 0.10 | |  | |  | |  |
| Widow/divorced |  | 0.87 [0.66 ; 1.14] | 0.3 | |  | | 1.66 [1.11 ; 2.50] | | 0.01 | |  | | 1.09 [0.83 ; 1.42] | | 0.6 | |  | |  | |  |

Characteristics are adjusted on each other.

**Suppl 6. Table 2: (Model 2) Multivariable associations (OR and 95% CI using multinomial logistic regression model) between socio-demographic and lifestyle characteristics and sub-phenotypes group membership.**

|  |  | **Sub-phenotype 2** | |  | **Sub-phenotype 3** | |  | **Sub-phenotype 4** | |  | *p*-value |
| --- | --- | --- | --- | --- | --- | --- | --- | --- | --- | --- | --- |
|  |  |  |  |  |  |  |  |  |  |  |  |
| N=3398 |  | OR (95% CI) | *p*-value |  | OR (95% CI) | *p*-value |  | OR (95% CI) | *p*-value |  |  |
|  |  |  |  |  |  |  |  |  |  |  |  |
| Age, years |  | 1.07 [1.05 ; 1.09] | <0.0001 |  | 1.09 [1.07 ; 1.12] | <0.0001 |  | 1.08 [1.06 ; 1.10] | <0.0001 |  | <0.0001 |
|  |  |  |  |  |  |  |  |  |  |  |  |
| Sex |  |  |  |  |  |  |  |  |  |  | <0.0001 |
| Men |  | 0.83 [0.67 ; 1.03] | 0.08 |  | 0.80 [0.56 ; 1.12] | 0.2 |  | 1.52 [1.22 ; 1.91] | <0.0001 |  |  |
| Women |  | 1 (ref) |  |  | 1 (ref) |  |  | 1 (ref) |  |  |  |
|  |  |  |  |  |  |  |  |  |  |  |  |
| Ethnicity |  |  |  |  |  |  |  |  |  |  | 0.05 |
| Caucasian |  | 1 (ref) |  |  | 1 (ref) |  |  | 1 (ref) |  |  |  |
| Non-Caucasian |  | 1.06 [0.70 ; 1.58] | 0.8 |  | 1.11 [0.59 ; 2.00] | 0.7 |  | 1.57 [1.07 ; 2.31] | 0.02 |  |  |
|  |  |  |  |  |  |  |  |  |  |  |  |
| Marital status |  |  |  |  |  |  |  |  |  |  | 0.003 |
| Single |  | 0.86 [0.65 ; 1.14] | 0.3 |  | 1.44 [0.94 ; 2.11] | 0.09 |  | 0.69 [0.51 ; 0.92] | 0.013 |  |  |
| Married/cohabitant |  | 1 (ref) | 0.3 |  | 1 (ref) | 0.06 |  | 1 (ref) | 0.03 |  |  |
| Widow/divorced |  | 0.83 [0.63 ; 1.10] | 0.2 |  | 1.54 [1.02 ; 2.33] | 0.03 |  | 1.02 [0.78 ; 1.34] | 0.8 |  |  |
|  |  |  |  |  |  |  |  |  |  |  |  |
| Alcohol consumption |  |  |  |  |  |  |  |  |  |  | 0.002 |
| No in the previous week |  | 1 (ref) | **0.002^b^** |  | 1 (ref) | 0.2 |  | 1 (ref) | **0.0003** |  |  |
| Moderate, 1-14 units/wk |  | 0.72 [0.57 ; 0.92] | 0.009 |  | 0.73 [0.50 ; 1.07] | 0.1 |  | 0.64 [0.50 ; 0.81] | <0.0001 |  |  |
| High, >14 units/wk |  | 1.00 [0.75 ; 1.35] | 0.9 |  | 0.72 [0.43 ; 1.20] | 0.2 |  | 0.84 [0.62 ; 1.12] | 0.2 |  |  |
|  |  |  |  |  |  |  |  |  |  |  |  |
| Fruits and vegetables intake | | |  |  |  |  |  |  |  |  |  |
| Less than one daily |  | 1.49 [1.17 ; 1.90] | 0.001 |  | 1.65 [1.13 ; 2.41] | 0.01 |  | 1.60 [1.26 ; 2.03] | <0.0001 |  |  |
| At least once daily |  | Ref |  |  | 1 (ref) |  |  | 1 (ref) |  |  |  |
|  |  |  |  |  |  |  |  |  |  |  |  |
| Smoking status |  |  |  |  |  |  |  |  |  |  | 0.17 |
| Non-smoker |  | 1 (ref) | 0.3 |  | 1 (ref) | 0.3 |  | 1 (ref) | **0.03** |  |  |
| Former smoker |  | 1.11 [0.92 ; 1.33] | 0.2 |  | 1.26 [0.92 ; 1.71] | 0.1 |  | 1.26 [1.05 ; 1.52] | 0.01 |  |  |
| Current smoker |  | 0.78 [0.44 ; 1.37] | 0.4 |  | 1.31 [0.57 ; 3.08] | 0.5 |  | 0.89 [0.51 ; 1.56] | 0.7 |  |  |
|  |  |  |  |  |  |  |  |  |  |  |  |
| Sedentarily behavior |  |  |  |  |  |  |  |  |  |  | 0.11 |
| High |  | 1.19 [0.97 ; 1.47] | 0.09 |  | 1.24 [0.87 ; 1.78] | 0.2 |  | 1.39 [1.13 ; 1.72] | 0.002 |  |  |
| Moderate |  | 1.08 [0.87 ; 1.35] | 0.4 |  | 1.23 [0.85 ; 1.78] | 0.2 |  | 1.20 [0.96 ; 1.49] | 0.1 |  |  |
| Low |  | 1 (ref) | 0.25 |  | 1 (ref) | 0.4 |  | 1 (ref) | **0.008** |  |  |
|  |  |  |  |  |  |  |  |  |  |  |  |

Characteristics are adjusted on each other.

^b^Test for difference.

# Supplemental 7: Distribution of clinical combination of MetS, Pre-frailty and MetS-Pre-frailty co-occurrence

**Combinations of metabolic syndrome criteria in the Whitehall II cohort**

The overall prevalence of MetS in the study population was 44.8%, with a higher proportion observed in men than in women (46.9% vs 38.9%, p < 0.0001). Among the five MetS components, the most frequent criterion was hypertension (64.6%), followed by hypertriglyceridemia (49.4%), low HDL-cholesterol levels (43.7%), and abdominal obesity (42.0%). Fasting hyperglycemia (25.2%) was the least prevalent component.

The analysis of clinical criterion combinations identified all sixteen theoretically possible MetS configurations (i.e., all combinations of three, four, or five criteria among the five MetS components), and all were observed in the study population.

As shown in Suppl 7. Figure 1, the distribution of metabolic profiles was highly heterogeneous. Two main combinations accounted for nearly half of all cases: “low HDL-cholesterol – hypertriglyceridemia – hypertension” (24.9%) and “low HDL-cholesterol – abdominal obesity – hypertriglyceridemia – hypertension” (22.1%). The full MetS profile including all five criteria (“low HDL-cholesterol – abdominal obesity – fasting hyperglycemia – hypertriglyceridemia – hypertension”) accounted for 17.3% of cases. This was followed by a four-component profile characterized predominantly by lipid and blood pressure abnormalities (“low HDL-cholesterol – fasting hyperglycemia – hypertriglyceridemia – hypertension”) (10.6%), and by a three-component profile combining obesity, hyperglycemia, and hypertension (6.5%). The remaining eleven combinations were rare, each representing less than 5% of cases.

**Suppl 7. Figure 1: Combinations of metabolic syndrome criteria in the Whitehall II cohort (participants with MetS, n = 1,523)**

**Combinations of pre-frailty criteria in the Whitehall II cohort**

The overall prevalence of pre-frailty (defined as the presence of one or two Fried criteria out of five) was estimated at 47.3% in the study population. Women showed a significantly higher prevalence than men (50.8% vs 46.0%, p = 0.01). Among the five components of the Fried phenotype, the most frequent in the population were low physical activity (26.6%) and low handgrip strength (22.1%), followed by exhaustion (6.5%), unintentional weight loss (2.5%), and slow walking speed (2.2%).

There are 15 theoretically possible configurations of pre-frailty criterion combinations, corresponding to all combinations of one or two positive criteria among the five Fried components. Among these, 14 combinations were observed in the population. The dominant profiles were largely driven by single isolated criteria, particularly low physical activity (34.3%) and low handgrip strength (27.1%), which together accounted for more than 60% of pre-frailty cases. The combination of low handgrip strength and low physical activity ranked third (15.6%), followed by exhaustion alone (7.2%). The remaining combinations were rare, each representing less than 5% of cases.

These results highlight a strong asymmetry in the distribution of pre-frailty combinations, largely dominated by low physical activity and reduced muscular strength, which represent the most frequent pre-frailty profiles (34.3% and 27.1%, respectively). The combination of these two criteria also constituted a substantial profile (15.6%), suggesting that reduced physical activity and diminished grip strength form the core pattern of pre-frailty in this population. Other combinations were considerably less frequent, particularly those involving exhaustion (7.2% alone; 3.9% when combined with inactivity), while profiles including unintentional weight loss or slow walking speed remained marginal (≤3%). Overall, these findings indicate that, in the studied population, pre-frailty is primarily expressed through early motor and behavioral impairments, preceding the emergence of more advanced manifestations related to energy balance, nutrition, or mobility.

Suppl. 7 Figure 2: Combinations of pre-frailty criteria in the Whitehall II cohort (pre-frail participants, n = 1,606)

#### Clinical combinations of MetS-prefrailty co-occurrence

The overall prevalence of MetS–pre-frailty co-occurrence in the Whitehall II cohort was estimated at 23.2%, with a slightly higher prevalence in men than in women (23.9% vs 21.3%, p < 0.0001).

To better characterize the clinical diversity of this dual condition, a combinatorial analysis was conducted by cross-classifying the 16 identified MetS combinations with the 14 pre-frailty combinations, resulting in 224 theoretically possible combinations. Among these, 104 distinct combinations were observed in the study population, reflecting substantial heterogeneity in co-occurring metabolic and functional profiles. Suppl 7. Figure 3 presents the 24 most prevalent combinations (≥1%), which together account for the majority of observed cases. These profiles correspond to the most frequent clinical configurations and illustrate the complexity of the joint expression of MetS and pre-frailty.

The results highlight a strong predominance of combinations involving both lipid abnormalities (low HDL-cholesterol and elevated triglycerides) and hypertension, often co-occurring with functional impairments such as low physical activity or reduced handgrip strength. The three most frequent combinations—(i) low physical activity + low HDL-cholesterol + elevated triglycerides + hypertension (8.1%), (ii) low physical activity + abdominal obesity + low HDL-cholesterol + elevated triglycerides + hypertension (7.2%), and (iii) low physical activity + abdominal obesity + low HDL-cholesterol + fasting hyperglycemia + elevated triglycerides + hypertension (6.7%)—together accounted for more than 20% of all cases.

Combinations involving fasting hyperglycemia or exhaustion were less frequent (approximately 1–2%), while those including unintentional weight loss were rare. Overall, these findings suggest that MetS–pre-frailty co-occurrence is predominantly characterized by a phenotype combining lipid dysregulation, hypertension, and reduced physical performance, reflecting a systemic vulnerability that integrates metabolic dysfunction and early functional decline.

**Suppl 7. Figure 3: Combinations of clinical criteria for the co-occurrence of MetS and pre-frailty in the Whitehall II cohort.**

# Supplemental 8: Internal validation metrics for optimal cluster selection (k = 2 to 10).

**Suppl 8. Table 1 Internal validation metrics across candidate cluster solutions (k = 2 to 10).**

| **k** | **Calinski-Harabasz Index** | **Total Within-Cluster Sum of Squares (WSS)** | **Average Silhouette Width** |
| --- | --- | --- | --- |
| 2 | 370.13 | 30 631.47 | 0.0936 |
| 3 | 336.41 | 28 351.30 | 0.1190 |
| **4** | **377.58** *(Peak)* | **25 469.51** *(Elbow)* | **0.1352** |
| 5 | 369.60 | 23 660.53 | 0.1466 *(Peak)* |
| 6 | 350.81 | 22 391.22 | 0.1358 |
| 7 | 320.03 | 21 688.68 | 0.1311 |
| 8 | 308.07 | 20 762.47 | 0.1358 |
| 9 | 290.02 | 20 164.94 | 0.1142 |
| 10 | 273.14 | 19 686.28 | 0.0884 |
